# Pregabalin Use in Treatment-Resistant Depression: A Case Report

**DOI:** 10.7759/cureus.83301

**Published:** 2025-05-01

**Authors:** Devon A McCoy, Sadie R Walter, Matthew Bogoyas

**Affiliations:** 1 Psychiatry, Elson S. Floyd College of Medicine, Spokane, USA; 2 Psychiatry, Pacific Northwest University of Health Sciences, Yakima, USA; 3 Psychiatry, Elson S. Floyd College of Medicine, Tri-Cities, USA

**Keywords:** anticonvulsants in psychiatry, augmentation therapy, gaba modulation, glutamate regulation, major depressive disorder, personalized medicine in mental health, pharmacological mechanisms, pregabalin, treatment-resistant depression

## Abstract

Treatment-resistant depression (TRD) significantly contributes to morbidity and mortality, characterized by persistent depressive symptoms despite comprehensive therapeutic interventions. Standard antidepressants for managing major depressive disorder (MDD) primarily target the serotonin, dopamine, and norepinephrine pathways. Pregabalin, an anxiolytic anticonvulsant, shows promise as an adjunct therapy for TRD. This case report details a 57-year-old male with a 10-year history of MDD who experienced substantial symptom improvement following the addition of pregabalin to his treatment regimen. Previous interventions, including traditional antidepressants, antipsychotics, thyroid supplementation, stimulants, stimulant-like medications, electroconvulsive therapy, and transcranial magnetic stimulation, were either poorly tolerated or ineffective. This case demonstrates pregabalin's potential as an effective adjunct in TRD management and underscores the importance of individualized treatment strategies to optimize outcomes for patients with complex psychiatric and medical needs.

## Introduction

Major depressive disorder (MDD) is a prevalent and debilitating mental health condition, affecting an estimated 21 million adults annually in the United States [1]. It is characterized by episodes of depressed mood and anhedonia lasting at least two weeks. Standard treatment typically involves initiation of psychotherapy, alongside a combination of pharmacological interventions, such as selective serotonin reuptake inhibitors (SSRIs) and serotonin-norepinephrine reuptake inhibitors (SNRIs) [2]. Despite these therapies, approximately 30% of patients develop treatment-resistant depression (TRD), typically defined as inadequate symptom resolution following at least two antidepressant trials at adequate dose and duration [3]. The mechanisms underlying TRD are not fully understood but may involve factors such as early-life trauma, chronic stress, and hypothalamic-pituitary-adrenal (HPA) axis dysregulation [4].

Pregabalin, a gamma-aminobutyric acid (GABA) analog anticonvulsant, is approved for neuropathic pain and is frequently used off-label for generalized anxiety disorder (GAD). It acts on voltage-gated calcium channels by binding to the α_2_δ subunit, reducing neurotransmitter release [5]. Although its role in TRD is not well-studied, its ability to modulate cortical GABA deficits and glutamate imbalances associated with depression suggests a potential role in mood regulation [6,7]. This case highlights pregabalin’s potential therapeutic value in TRD management through its unique mechanism of action.

## Case presentation

A 57-year-old male presented with a 10-year history of MDD, characterized by depressed mood, anxiety, insomnia, anhedonia, avolition, and intermittent suicidal ideation (SI). He was treated in a community outpatient setting at the time of presentation, but had been hospitalized twice in the past four years due to SI. His medical history included obesity, hypertension, hyperlipidemia, and degenerative joint disease. Family psychiatric history was notable for paternal suicide and a sister possibly diagnosed with bipolar disorder. The patient had previously received a diagnosis of bipolar II disorder, although clinical documentation lacked evidence of clear manic or hypomanic features. He also reported multiple adverse childhood experiences (ACEs), including discovering his father’s suicide.

At the time of presentation, the patient experienced significant functional impairment, requiring assistance with basic activities of daily living and rarely leaving the home. His social interactions were largely limited to immediate family, with a marked reduction in quality of life. A mental status examination at presentation revealed anxious mood, restricted affect, coherent thought processes, and fair insight and judgment. The patient’s Patient Health Questionnaire-9 (PHQ-9) score was 25, consistent with severe depressive symptoms. Physical and neurological examinations were unremarkable. Laboratory assessments showed no acute abnormalities. Potential medical contributors to psychiatric symptoms were considered and ruled out through physical examination, laboratory testing, and clinical evaluation.

The patient underwent multiple medication trials, including SSRIs, SNRIs, mood stabilizers, stimulants, and antipsychotics (Table 1). Venlafaxine initially provided symptom relief, but exacerbated his anxiety at higher doses. Duloxetine and citalopram also led to temporary symptom improvement, but lost effectiveness over time. Depakote produced mild symptom relief but caused significant tremors. Non-pharmacological interventions included 15 sessions of electroconvulsive therapy (ECT), which provided mild improvement in SI but had minimal impact on other depressive symptoms. The patient was unable to tolerate transcranial magnetic stimulation (TMS) due to discomfort during the initial session. During a 2020 psychiatric hospitalization, the patient engaged in eye movement desensitization and reprocessing (EMDR) therapy, which led to partial improvement in depressive symptoms, particularly those related to trauma. He was also referred to outpatient psychotherapy, including EMDR therapy and individual counseling, and was encouraged to use a therapy workbook for additional support between sessions, but engagement was limited.

**Table 1:** Summary of treatments and outcomes in a 57-year-old male with treatment-resistant depression (TRD) The table summarizes the patient’s pharmacological and non-pharmacological treatment history, highlighting key medication classes, effects, side effects, and continuation status relevant to treatment resistance.

Category	Treatment Type	Effectiveness	Side Effects	Continuation Status
Antidepressants	Venlafaxine (SSRI)	Transient improvement	Increased anxiety at higher doses	Discontinued
	Duloxetine (SNRI)	Initial benefit, later ineffective	Increased anxiety at higher doses	Discontinued
	Citalopram (SSRI)	Initial benefit, later ineffective	None reported	Discontinued
Mood Stabilizers	Depakote (Valproic Acid)	Moderate symptom relief initially	Tremor	Discontinued
Antipsychotics	Zyprexa (Olanzapine)	Minimal symptom improvement	Sedation, weight gain	Discontinued
	Risperidone	Minimal symptom improvement	None reported	Discontinued
Stimulants	Modafinil	Mild symptom improvement	Insomnia	Discontinued
	Armodafinil	Improved engagement	Increased anxiety, poor hygiene	Discontinued
	Adderall	Improved engagement	Increased anxiety	Discontinued
Other Pharmacologic	Levothyroxine	No improvement	Increased anxiety, poor hygiene	Discontinued
	Pregabalin	Significant improvement in mood, anxiety, and tremor	Transient lightheadedness (resolved with amitriptyline dose adjustment)	Continued
	Auvelity (Dextromethorphan/Bupropion)	Partial improvement without adjunct	None reported	Continued
	Propranolol	Partial improvement without adjunct	None reported	Continued
	Amitriptyline	Partial improvement without adjunct	Lightheadedness (resolved with dose adjustment)	Continued
Non-Pharmacologic	ECT (Electroconvulsive Therapy)	Mild improvement in SI, minimal effect on mood	Minimal	Discontinued
	TMS (Transcranial Magnetic Stimulation)	Not tolerated	Discomfort	Discontinued
	EMDR (Eye Movement Desensitization and Reprocessing) Therapy	Partial improvement in trauma-related depressive symptoms	Minimal	Discontinued

Since entering our care, the patient was prescribed Auvelity (dextromethorphan/bupropion), amitriptyline, and propranolol. However, he continued to experience a lack of motivation, low energy, and fatigue. Suspecting that some of his medications were potentially contributing to these symptoms, we initiated a trial of 100 mg of modafinil, aiming to reduce or discontinue some of his existing medications while also addressing his TRD. The patient was instructed to monitor his anxiety levels. When no change occurred, the dose was doubled, resulting in mild improvement in disposition but causing intolerable insomnia. Alternatives, including armodafinil and Adderall, were trialed. Although the patient became more active with his family, he reported worsening anxiety, poor hygiene, and persistent anhedonia.

After these interventions failed, levothyroxine was trialed off-label as an adjunctive treatment for refractory depressive symptoms, which was discontinued after no improvements were noted. Pregabalin was then initiated at 50 mg once daily and later titrated to 75 mg once daily as tolerated to address the patient's persistent depressive symptoms. At his three-week follow-up, he reported mild mood improvement, though he experienced transient lightheadedness, likely due to an interaction with amitriptyline. This side effect resolved after reducing the amitriptyline dose. The patient continued on a regimen of pregabalin 75 mg once daily, amitriptyline 25 mg twice daily, propranolol XL 120 mg once daily, and Auvelity 45 mg/105 mg twice daily.

At eight weeks, both the patient and his wife reported continued mood and anxiety improvements, alongside increased engagement in household activities. A clinical examination revealed decreased restlessness. The pregabalin dose was then titrated to 75 mg per day, and at his next appointment four weeks later, he reported significant improvement in mood and anxiety, with only residual hand-wringing as a physical manifestation of his restlessness. Following treatment adjustments, the patient’s PHQ-9 score improved from 25 to 14, indicating a shift from severe to moderate depression.

Since initiating pregabalin, the patient has demonstrated sustained improvement in depressive symptoms and anxiety. Early signs of restlessness, including difficulty sitting still during appointments and frequent pacing, have largely resolved. He now remains seated throughout appointments, with only mild hand-wringing observed. While he does not yet describe his mood as “happy,” his functional engagement has markedly increased. He was even able to attend his family’s large Christmas party, and the improvement in his mood and functionality is notable.

## Discussion

MDD is a widespread condition affecting millions worldwide. In 2021, an estimated 21 million adults in the United States reported experiencing at least one depressive episode in their lifetime [1]. A prospective observational study found that remission occurred in only 37% of 3,671 patients after initial treatment with citalopram, a commonly prescribed SSRI [8]. While subsequent treatment strategies increased remission rates to 67%, relapse rates and treatment failures became more frequent with each successive therapeutic trial.

Although there is no universal consensus on TRD criteria, it is commonly characterized by at least two unsuccessful trials of antidepressant pharmacotherapy in adequate dose and duration [3]. By this definition, our patient’s presentation aligns with TRD, as his depressive symptoms did not respond to multiple classes of medications, ECT, and TMS.

This case presents a 57-year-old male with TRD symptoms that have remained refractory to various therapeutic interventions. Despite a previous diagnosis of bipolar II disorder and a self-reported family history of bipolar disorder, the patient’s presentation lacked distinct manic or hypomanic features. Instead, his depressive symptoms improved with EMDR therapy during his 2020 hospitalization, which is typically utilized in the treatment of post-traumatic stress disorder. This positive response to EMDR, coupled with ineffective trials of mood stabilizers and antipsychotics, supports the conclusion that his symptoms are more consistent with TRD rather than bipolar II disorder. In addition to these psychiatric factors, his overweight status likely contributed to reduced physical activity, further exacerbating depressive symptoms and functional impairment.

**Figure 1:**
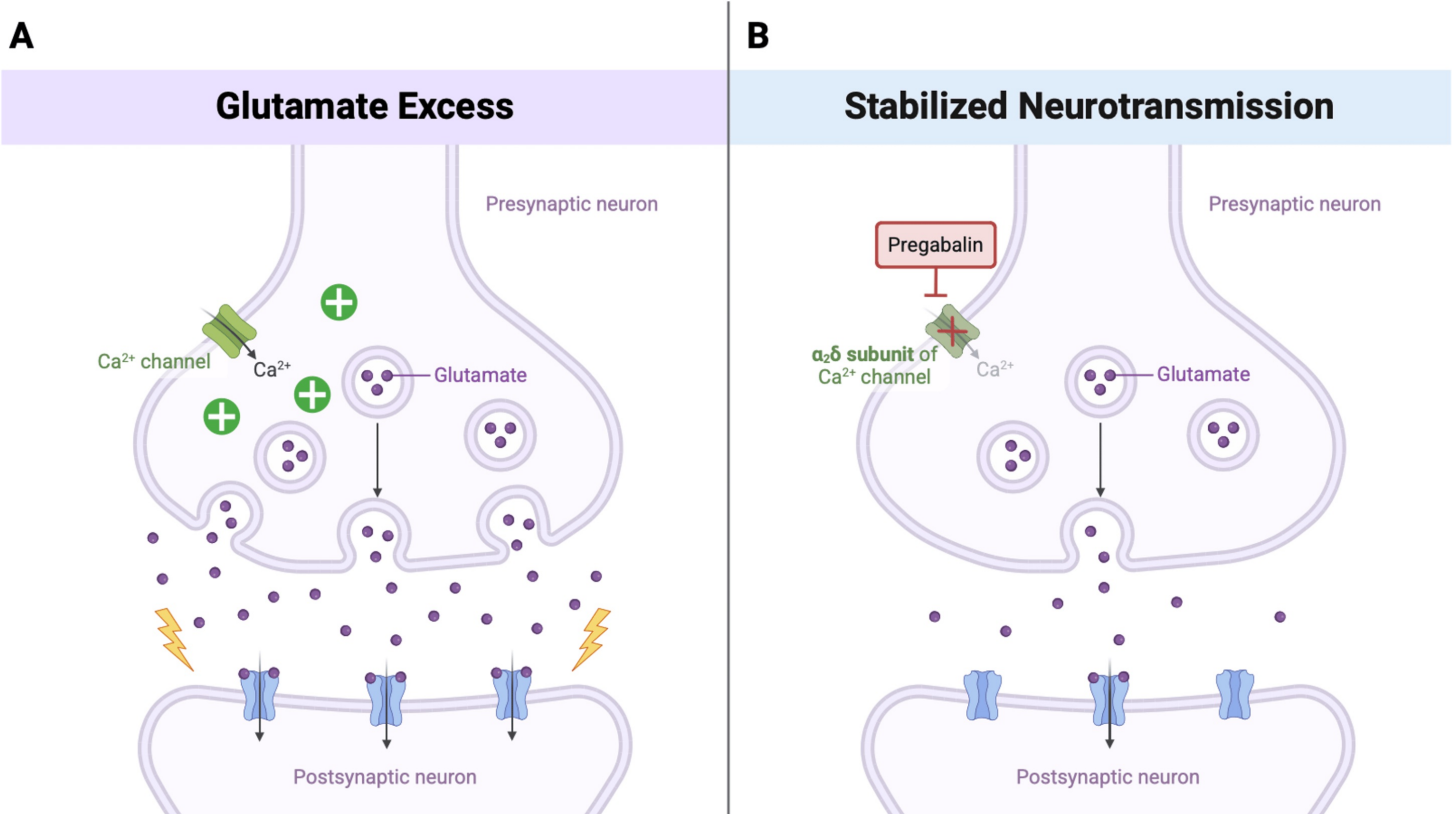
Mechanism of action of pregabalin in treatment-resistant depression 1A: Excess glutamate release due to uninhibited calcium channel activity in the presynaptic neuron contributes to glutamatergic hyperactivity. 1B: Pregabalin binds to the α_2_δ subunit of the calcium channel, stabilizing excitatory neurotransmission through reduced calcium influx. Image Credits: Created by Sadie Walter using BioRender

Pregabalin, primarily indicated for epilepsy and neuropathic pain, is also prescribed off-label for GAD in the United States and is formally approved to treat anxiety disorders in the European Union and the United Kingdom [9,10]. Pregabalin reduces excitatory neurotransmitter release through its effect on neuronal calcium channels, potentially mitigating the glutamatergic hyperactivity associated with depressive states (Figures 1A-1B) [6,7]. Additionally, its ability to increase GABA transporter protein density and functional transport could suggest a role in addressing the cortical GABA deficits implicated in unipolar depression [7]. By binding to the α2δ subunit, pregabalin reduces calcium influx at nerve terminals, decreasing glutamate release and indirectly enhancing GABAergic inhibitory transmission, contributing to the stabilization of neural activity.

A cross-sectional study of a European multicenter health service found that 7.23% of patients with MDD were concomitantly treated with pregabalin [11]. These patients exhibited severe, treatment-refractory symptoms, comorbid anxiety, and somatic manifestations. Our patient’s presentation aligns with this study’s patient cohort, particularly given his persistent depressive symptoms resistant to standard therapies alongside significant anxiety. These findings suggest that pregabalin may be beneficial in the management of complex TRD cases where standard treatments have failed, especially in patients whose comorbid anxiety exacerbates symptom severity. However, this study did not demonstrate a statistically significant difference in efficacy between pregabalin-containing regimens and other combination treatments, underscoring the need for further investigation into whether particular patient populations may find greater benefit from the use of pregabalin.

Prior to the introduction of pregabalin, the patient’s anxiety was severe and persistent. He routinely paced around the room, was unable to sit still, and his restlessness often prevented him from eating and drinking normally. Following pregabalin treatment, he has consistently demonstrated significant functional improvement - he has been able to remain seated throughout his appointments and reports reduced anxiety. At initial presentation, the patient endorsed severe depressive symptoms, reflected by a PHQ-9 score of 25, and persistent suicidal ideation. Following treatment, his PHQ-9 improved to 14, and suicidal ideation resolved. While some depressive symptoms persisted, improvements were noted in daily activity levels, ability to engage in limited social interactions, and resolution of passive suicidal ideation. This case highlights pregabalin’s potential synergistic effects when combined with other antidepressants, possibly due to its role in GABA and glutamate regulation [6,7].

## Conclusions

TRD management necessitates collaboration, patience, and persistence from both patients and clinicians, underscoring the importance of an individualized treatment approach. These findings highlight pregabalin’s potential role as a novel adjunct in TRD management, particularly in patients unresponsive to multiple treatment modalities. In this case, pregabalin was associated with notable reductions in depressive severity, resolution of suicidal ideation, and improved functional capacity. This patient’s substantial symptom improvement and increased engagement in daily activities suggest that pregabalin may be especially useful for addressing refractory symptoms in patients with co-occurring anxiety.

Despite these findings, large-scale clinical trials are needed to validate pregabalin’s role and clinical applicability in TRD management. Future research should target pregabalin’s long-term safety, efficacy, and identification of patient populations most likely to benefit from its use.

## References

[1] Substance Abuse and Mental Health Services Administration (2022). Key Substance Use and Mental Health Indicators in the United States: Results from the 2021 National Survey on Drug Use and Health (HHS Publication No. PEP22-07-01-005, NSDUH Series H-57). Key Substance Use and Mental Health Indicators in the United States: Results from the 2021 National Survey on Drug Use and Health (HHS Publication No. PEP22-07-01-005, NSDUH Series H-57).

[2] Bains N, Abdijadid S (2023). Major depressive disorder. StatPearls.

[3] Voineskos D, Daskalakis ZJ, Blumberger DM (2020). Management of treatment-resistant depression: challenges and strategies. Neuropsychiatr Dis Treat.

[4] Akil H, Gordon J, Hen R (2018). Treatment resistant depression: a multi-scale, systems biology approach. Neurosci Biobehav Rev.

[5] Taylor CP, Angelotti T, Fauman E (2007). Pharmacology and mechanism of action of pregabalin: the calcium channel α2-δ (alpha2-delta) subunit as a target for antiepileptic drug discovery. Epilepsy Res.

[6] Krystal JH, Sanacora G, Blumberg H (2002). Glutamate and GABA systems as targets for novel antidepressant and mood-stabilizing treatments. Mol Psychiatry.

[7] Frampton JE, Foster RH (2006). Pregabalin: in the treatment of generalised anxiety disorder. CNS Drugs.

[8] Rush AJ, Trivedi MH, Wisniewski SR (2006). Acute and longer-term outcomes in depressed outpatients requiring one or several treatment steps: a STAR*D report. Am J Psychiatry.

[9] Druschky K, Bleich S, Grohmann R (2018). Use and safety of antiepileptic drugs in psychiatric inpatients-data from the AMSP study. Eur Arch Psychiatry Clin Neurosci.

[10] Baldwin DS, Ajel K, Masdrakis VG, Nowak M, Rafiq R (2013). Pregabalin for the treatment of generalized anxiety disorder: an update. Neuropsychiatr Dis Treat.

[11] Dold M, Bartova L, Fugger G (2022). Pregabalin augmentation of antidepressants in major depression - results from a European multicenter study. J Affect Disord.

